# Perceived Academic Stress and Its Correlates Among Secondary School Adolescents in Kathmandu, Nepal

**DOI:** 10.1002/puh2.70186

**Published:** 2026-01-16

**Authors:** Rajesh Karki, Anita Tamang, Maheshor Kaphle

**Affiliations:** ^1^ Central Department of Public Health Institute of Medicine Tribhuvan University Kathmandu Nepal; ^2^ Department of Public Health, Yeti Health Science Academy, Purbanchal University, Maharajgunj Kathmandu Nepal; ^3^ Department of Public Health, People's Dental College and Hospital Tribhuvan University Kathmandu Nepal

**Keywords:** academic stress, adolescents, mental health, Nepal, students

## Abstract

**Background:**

Academic stress, defined as failure to meet academic demands despite adaptation efforts, is a global concern. It affects an estimated 10%–30% of students, compromising academic performance, psychological well‐being, and overall health.

**Aim:**

To assess perceived academic stress and its correlates among secondary school adolescents in Kathmandu, Nepal.

**Methods:**

In 2024, a cross‐sectional study was conducted in six selected private secondary schools in Kathmandu Metropolitan City, Nepal. A total of 353 students participated in this study. Perceived academic stress was measured using the validated Perception of Academic Stress (PAS) scale via self‐administered questionnaires. Data analysis employed descriptive statistics, one‐way analysis of variance (ANOVA), and independent sample *t*‐tests, with statistical significance set at *p* < 0.05. Normality was assessed using the Shapiro–Wilk test and *Q*–*Q* plots.

**Results:**

The prevalence of high academic stress was 46.7% (95% confidence interval [CI]: 41.4%–52.1%). The overall mean stress score was 52.58 ± 8.87 (range: 22.00–81.00). Among the PAS subscales, faculty workload and examinations had the highest mean score (22.22 ± 4.77). Male students reported significantly higher stress levels than female students (54.25 ± 8.25; *t* = 4.43, *p* < 0.001). Counterintuitively, students who reported not being worried about their grades had significantly higher stress scores than those who were worried (58.42 ± 8.88; *t* = 7.09, *p* < 0.001).

**Conclusion:**

Academic stress was highly prevalent among adolescents. Mitigation strategies should include adjusting faculty workload and examination demands, providing adequate preparation time, offering career guidance and realistic goal‐setting support, and enhancing social support systems.

## Graphical abstract

Academic stress is a prevalent public health challenge among adolescents in Kathmandu, with 46.7% reporting high levels. This study identified faculty workload and examinations as the primary sources of stress. The strategies should address workload, examinations, preparation time, career guidance, and social support.

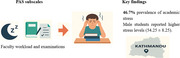

## 1 Introduction

Stress is a reaction to a situation that makes a person tired, irritable, and unable to enjoy normal life [1]. Academic stress, a specific form of stress, arises when students struggle to meet academic demands despite their efforts to adapt [2]. Perceived academic stress refers to students’ subjective experience of stress due to academic pressures such as heavy workloads, time constraints, and self‐evaluation of their academic abilities [3]. It is commonly associated with cognitive symptoms (e.g., negative thoughts), physiological responses (e.g., sleep disturbances, elevated heart rate), and motivational issues (e.g., loss of interest in academic tasks) [2].

Students are particularly susceptible to academic stress [4]. Academic pressure is a major source of distress among school‐aged children and adolescents [5]. Contributing factors include excessive academic workload, pressure to achieve high grades, unrealistic expectations, feelings of hopelessness, and the burden of numerous assignments [6]. Although early research identified parents as a source of academic stress due to high expectations, more recent studies highlight the dual role of families as both potential stressors and crucial support systems in mitigating stress [7].

Globally, an estimated 10%–30% of students experience academic stress, which can significantly affect their academic performance, psychosocial development, and overall health [8]. A large‐scale study by the Organization for Economic Co‐operation and Development (OECD), involving 540,000 students aged 15–16 across 77 countries, found that 66% of students were stressed about poor grades, 59% felt anxious about difficult exams, 55% experienced test anxiety, and 37% felt tense while studying [9]. In Ethiopia, 52.2% of high school students reported experiencing academic stress [10].

This trend is also evident in developing countries [10]. In Karnataka, India, 28% of Grade 11 and 26% of Grade 12 students reported high or extreme levels of academic stress [11]. In Bangladesh, the prevalence of stress symptoms among adolescents was found to be 73.5% [12]. Similarly, Nepal faces a growing mental health burden among its youth. Studies have indicated that one in four Nepali high school students experiences academic stress [13]. Although national‐level data are lacking, recent studies in Hetauda and Kathmandu have reported academic stress prevalence rates of 51.7% and 89.4%, respectively, among adolescent students [13, 14].

Academic stress has far‐reaching consequences. It is associated with anxiety, depression, poor academic outcomes, substance use, sleep disturbances, and unhealthy lifestyle behaviors. Over time, academic stress may also contribute to chronic noncommunicable diseases such as obesity, metabolic syndrome, and insulin resistance [15]. Alarmingly, academic failure has been linked to increased suicide risk among adolescents, particularly during examination periods [5].

Although several studies in Nepal have explored academic stress among college students, research focusing on adolescents and secondary school students remains limited [16]. Addressing this gap is important when creating targeted interventions. This study assessed the prevalence of perceived academic stress and identified key sociodemographic and academic correlates among secondary school adolescents in Kathmandu, Nepal. The findings will inform stakeholders, including schools, parents, and policymakers, about modifiable factors, such as workload management and support systems, to help mitigate academic stress in this vulnerable population.

## 2 Materials and Methods

### 2.1 Study Design, Setting, and Participants

A descriptive cross‐sectional study was conducted between April 30 and May 15, 2024, among secondary‐level students (Grades 8, 9, and 10; aged 10–19 years) in Ward 26 of Kathmandu Metropolitan City, Nepal. Six private English‐medium schools were selected from the 21 secondary schools in the ward based on administrative feasibility (approval timelines and accessibility) and resource constraints (time and budget). All students in Grades 8–10 at these six schools were eligible to participate.

### 2.2 Sample Size and Sampling Method

No formal sample size calculation was performed because this study employed a census sampling approach within the selected schools. Due to the study's scope and logistical constraints, the aim was to include all eligible students in Grades 8–10 across the six selected private schools, totaling an eligible population of *N* = 408.

A final sample of 353 participants was obtained from the eligible population of *N* = 408, yielding a response rate of 86.5%. Nonparticipation (*n* = 55) was attributed to the following exclusions: absence during data collection (*n* = 30), declined assent (*n* = 10), lack of parental consent (*n* = 5), and incomplete questionnaires (>10% missing data; *n* = 10). The initial selection of the six schools used convenience sampling based on administrative feasibility; however, all students were invited to participate.

### 2.3 Data Collection Tool and Variable Measurement

Data were collected using a structured, self‐administered English‐language questionnaire (the medium of instruction in all selected schools). The questionnaire was structured into distinct sections.

1. Sociodemographic and academic/environmental variables: This section captures essential participant characteristics and context using items developed by the researchers based on a literature review specific to the context [13].
2. Sociodemographic characteristics: Age, sex, ethnicity, religion, family type, and parent occupation.
3. Academic and school environment: Previous academic performance (e.g., last grade score) and perceptions of the school environment.
4. Academic stress. Academic stress was measured using the Perception Scale Academic Stress (PAS), an 18‐item scale developed by Bedewy and Gabriel [17].
5. Structure: The scale comprises three subscales: academic expectations (four items), faculty workload and examinations (eight items), and academic self‐perception (six items) [17].
6. Scoring: Items are scored on a Likert scale (strongly agree to strongly disagree), yielding a continuous total score range of 18–90, with higher scores indicating higher stress levels [18, 19].
7. Categorization: For prevalence estimation, students were categorized as having high stress (≥54) or low stress (≤53) using a cutoff from prior studies [20]. Continuous scores were used for all inferential analyses.

### 2.4 Validity, Reliability, and Pilot Testing

The PAS has demonstrated validity and reliability in measuring academic stress [19, 21], including prior use in Nepal [20]. A pretest was conducted on 10% of the sample (*n* = 40) from a school in Ward No. 16 that was demographically similar. This confirmed the reliability of the tool (Cronbach's *α* = 0.8). Minor wording changes were made for clarity on the basis of the feedback of the participants. The pilot participants were excluded from the main analysis.

### 2.5 Data Collection Procedures

The school administration provided the approval and class schedules. Parental consent forms were distributed in advance. Written assent was obtained from the participants on the day of data collection. The researchers provided standardized verbal and written instructions. Each questionnaire item was read aloud, ambiguities were clarified, and examples were provided to ensure comprehension. Participants completed the questionnaire anonymously in classrooms. Students were initially given 1 day to return their completed questionnaire along with signed parental consent forms. Researchers revisited schools during the study period to collect questionnaires and consent forms to maximize participation. Daily quality checks were conducted to identify missing or incomplete responses. Following conventional practice, questionnaires with >10% missing data or incomplete consent forms were excluded from the analysis. Follow‐up efforts were made by contacting students during subsequent visits to resolve minor missing data (blank items). To maintain consistency, no new participants were enrolled during the follow‐up period.

### 2.6 Statistical Analysis

All data were coded and entered into the Statistical Package for the Social Sciences version 26 for analysis. Descriptive statistics, including frequencies, percentages, means, and standard deviations, were used to describe the characteristics and PAS scores of the participants. The prevalence of academic stress was calculated as the proportion of participants classified as having “high stress” based on a predefined cutoff, along with a 95% confidence interval (CI). Group differences in PAS scores were analyzed using one‐way analysis of variance (ANOVA) and independent samples *t*‐tests. Pearson's correlation analysis was conducted to assess the strength and direction of the relationships between the PAS subscales and total stress score. The normality of continuous variables was assessed using the Shapiro–Wilk test and *Q*–*Q* plots, whereas Levene's test was used to evaluate the homogeneity of variances. A significance level of 0.05 was set for all analyses.

## 3 Results

### 3.1 Sociodemographic Profile

The majority (67.4%) of the 353 respondents were in middle adolescence (14–17 years), with a mean age of 14.18 ± 1.20 years. Males were more prevalent (59.8%) than females. Most respondents identified themselves as Janajati (36.0%) and Hindu (77.1%), lived with their families (91.5%), and resided in nuclear families (67.7%). Parental education levels were predominantly secondary (53.0% for fathers, 41.6% for mothers), with fathers primarily engaged in business (41.6%) and mothers being homemakers (66.3%) (Table 1).

**TABLE 1:** Respondents’ sociodemographic characteristics (*n* = 353).

Sociodemographic characteristics	Frequency	Percentage
Age		
>Mean, SD	14.18 ± 1.200	
10–13 (early adolescence)	115	32.6
14–17 (middle adolescence)	238	67.4
Sex		
Male	211	59.8
Female	142	40.2
Ethnicity		
Brahmin	48	13.6
Chhetri	83	23.5
Janajati	127	36.0
Dalit	19	5.4
Madhesi	16	4.5
Others	60	17.0
Religion		
Hindu	272	77.1
Buddhist	57	16.1
Christian	9	2.5
Muslim	9	2.5
Others	6	1.7
Current grade		
Grade 8	114	32.3
Grade 9	104	29.5
Grade 10	135	38.2
Living arrangement		
Living with the family	323	91.5
Living with relatives	16	4.5
Living in a hostel	14	4.0
Types of family		
Nuclear	239	67.7
Joint	98	27.8
Extended	16	4.5
Father's education		
Unable to read and write	8	2.3
Can read and write but has no formal education	14	4.0
Basic level (1–8 grades)	80	22.7
Secondary level (9–12 grades)	187	53.0
University level	64	18.1
Mother's education		
Unable to read and write	32	9.1
Can read and write but has no formal education	20	5.7
Basic level (1–8 grades)	109	30.9
Secondary level (9–12 grades)	147	41.6
University level	45	12.7
Main occupation of father		
Agriculture	17	4.8
Business	147	41.6
Labor	19	5.4
Professionals	91	25.8
Others	79	22.4
Main occupation of mother		
Agriculture	10	2.8
Business	70	19.8
Labor	12	3.4
Professionals	27	7.6
Housemaker	234	66.3

### 3.2 Academic Performance, Extracurricular Preferences, and Perception of the School Environment

Most respondents (71.1%) reported achieving grades ranging from A+ to B+ on their previous examinations. However, most participants (77.6%) expressed concerns about their academic performance. Interestingly, most participants (69.7%) preferred physical‐related extracurricular activities (ECAs) to academic activities (30.3%). The respondents (50%) rated the school environment as good, whereas the other half (46%) rated it as moderate (Table 2).

**TABLE 2:** Respondents’ academic performance, extracurricular preferences, and school environment rating (*n* = 353).

Academic and school environment characteristics		
Previous academic grade	Frequency	Percentage
A+–B+	251	71.1
B–C+	90	25.5
C–D+	12	3.4
Worried about grades?		
Yes	274	77.6
No	79	22.4
Preferred extracurricular activities (ECAs)		
Academic‐related ECA	107	30.3
Physical‐related ECA	246	69.7
How do you rate the school environment?		
Good	177	50.1
Moderate	163	46.2
Bad	13	3.7

### 3.3 Academic Stress: Prevalence, Subscale Scores, and Correlations

The prevalence of academic stress was 46.7% (95% CI: 41.4%–52.1%), with nearly half of the respondents reporting high levels of stress. The mean score for the PAS scale was 52.58 ± 8.87 (range: 22.0–81.0). Among the subscales, the faculty workload and examinations subscale had the highest mean score (22.22 ± 4.77), followed by students’ academic self‐perceptions (19.52 ± 4.05) and academic expectations (10.84 ± 2.74), indicating that workload and exams were perceived as the most stressful (Table 3). Pearson's correlation analysis revealed strong, significant, and positive associations between all subscales and the total stress score. The strongest relationships were observed between faculty work and examinations (*r* = 0.833, *p* < 0.001) and between academic self‐perception and total stress score (*r* = 0.748, *p* < 0.001) (Table 4).

**TABLE 3:** Mean and standard deviation of the subscales.

Variable	Subscales	Min–Max	Mean ± SD
Academic stress	Academic expectation	4.00–20.00	10.84 ± 2.74
	Faculty work and examination	8.00–36.00	22.22 ± 4.78
	Academic self‐perception	7.00–30.00	19.52 ± 4.05

**TABLE 4:** Correlation between academic stress subscales and perceived academic stress scores.

Variable	Subscale	PAS score	*p* value	Relationship
Academic stress	Academic expectation	0.682**	<0.001	Strong positive correlation
	Faculty work and examination	0.833**	<0.001	Very strong positive correlation
	Academic self‐perception	0.748**	<0.001	Strong positive correlation

Abbreviation: PAS, Perception Scale Academic Stress.

** indicates statistically significant correlation at p <0.001 (two‐tailed).

### 3.4 Association Between Sociodemographic Factors and Academic Stress

Table 5 presents the results of the ANOVA and independent sample *t*‐tests, which examine the relationship between academic stress and various sociodemographic factors. The results indicated a statistically significant difference in academic stress according to sex (*t* = 4.432, *p* < 0.001). Male respondents reported higher levels of academic stress (*M* = 54.25, SD = 8.25) than female respondents (*M* = 50.09, SD = 9.21). However, no significant differences were found for age (*t* = −0.095, *p* = 0.924), religion (*t* = 1.628, *p* = 0.105), current grades (*F* = 2.179, *p* = 0.115), current living arrangements (*F* = 2.304, *p* = 0.101), family type (*F* = 0.011, *p* = 0.989), fathers’ education (*t* = 0.257, *p* = 0.798), mothers’ education (*t* = 0.094, *p* = 0.925), fathers' occupation (*F* = 0.579, *p* = 0.678), or mothers’ occupation (*F* = 0.397, *p* = 0.811).

**TABLE 5:** Association between sociodemographic factors and academic stress scores (*n* = 353).

Sociodemographic characteristics	Academic stress	Test statistics	*p* value
	**Mean** ± **SD**		
Age			
10–13 (early adolescence)	52.51 ± 9.61	*t* = −0.095	0.924
14–17 (middle adolescence)	52.61 ± 8.52		
Sex			
Male	54.25 ± 8.25	*t* = 4.432	<0.001
Female	50.09 ± 9.21		
Religion			
Hindu	52.99 ± 8.97	*t* = 1.628	0.105
Other than Hindus	51.17 ± 8.44		
Students’ grades			
Grade 8	53.18 ± 9.70		
Grade 9	53.52 ± 7.97	*F* = 2.179	0.115
Grade 10	51.34 ± 8.73		
Current living arrangements			
Living with the family	52.76 ± 8.98		
Living with relatives	48.00 ± 7.73	*F* = 2.304	0.101
Living in a hostel	53.57 ± 6.20		
Types of family			
Nuclear	52.55 ± 9.21		
Joint	52.60 ± 8.13	*F* = 0.011	0.989
Extended	52.88 ± 8.52		
Father education			
Illiterate	53.38 ± 7.39		
Illiterate	52.56 ± 8.91	*t* = 0.257	0.798
Mother education			
Illiterate	52.72 ± 6.95	*t* = 0.094	0.925
Literate	52.56 ± 9.05		
Main occupation of father			
Agriculture	54.18 ± 7.65		
Business	52.69 ± 8.07	*F* = 0.579	0.678
Labor	50.16 ± 12.34		
Professionals	52.22 ± 9.66		
Other	53.01 ± 8.73		
Main occupation of mother			
Agriculture	51.70 ± 4.14		
Business	52.69 ± 9.01		
Labor	52.58 ± 7.56	*F* = 0.397	0.811
Professionals	54.56 ± 10.42		
Housemaker	52.35 ± 8.88		

### 3.5 Association Between Academic Stress and School Environment

Table 6 presents the results of the independent sample *t*‐test and one‐way ANOVA. The results indicated a statistically significant difference in stress levels between respondents who worried about grades and those who did not (*t* = 7.090, *p* < 0.001). Students who did not worry about their grades reported significantly higher stress levels (*M* = 58.42, SD = 8.88) than those who did (*M* = 50.89, SD = 8.14). However, no significant differences were found in stress levels across grade categories (*F* = 1.722, *p* = 0.180), between the most preferred ECAs (*t* = 0.785, *p* = 0.433), or in school environment ratings (*F* = 2.293, *p* = 0.102) (Table 6).

**TABLE 6:** Association between academic stress score and academic/school environment characteristics (*n* = 353).

Academic and school environment characteristics	Academic stress	Test statistics	*p* value
	**Mean** ± **SD**		
Previous academic grade			
A+–B+	53.09 ± 9.13	*F* = 1.722	0.180
B–C+	51.54 ± 8.42		
C–D+	49.58 ± 5.11		
Worried about grades?			
Yes	50.89 ± 8.14	*t* = 7.090	<0.001
No	58.42 ± 8.88		
Preferred extracurricular activities (ECAs)			
Academic‐related ECA	53.14 ± 8.42	*t* = 0.785	0.433
Physical‐related ECA	52.33 ± 9.07		
How did you rate the school environment?			
Good/Very good	53.58 ± 8.99		
Moderate	51.58 ± 8.70	*F* = 2.293	0.102
Bad	51.38 ± 8.50		

## 4 Discussion

This community‐based cross‐sectional study assessed secondary school students’ academic stress in Kathmandu, Nepal. The mean academic stress score (52.58 ± 8.87) was consistent with that of a previous Nepali study [20]. Notably, 46.7% of the respondents reported high academic stress using the established cutoff, indicating a substantial burden. The prevalence rates across Nepal vary considerably, from 22.2% in Karnali Province [16], 27.5% in Kathmandu Metropolitan [22] to 34.4% in Pokhara [7], and higher rates of 51.67% in Hetauda [13] and 79.3% in Kathmandu [14]. Methodological differences (assessment tools, cutoffs), sample characteristics (age, stream), school environment variations, and sociocultural contexts are likely to explain these discrepancies. Stress levels also differ by academic level and field, with bachelor's level health science students reporting rates between 27.2% and 67.1% [23, 24]. Collectively, these findings reveal that academic stress is a pressing public health concern among Nepali adolescents, indicating the need for mental health support interventions that address academic‐related pressures in this population.

Faculty workload and examinations emerged as the most prominent perceived stressors in this context. This result aligns with previous studies conducted in Nepal [25, 26, 27]. The high levels of stress reported concerning curriculum demands, assignment volume, and examination pressures likely reflect underlying systemic issues within the educational environment, including rigid curricula, frequent high‐stakes assessments, and societal norms that strongly link academic achievement to future socioeconomic stability. Chronic exposure to these demands may impair mental health and academic performance. Positive parenting styles and strong social support from families, peers, and teachers can mitigate academic stress among students [2, 6].

Male students reported significantly higher stress levels than female students. This finding contrasts with some previous studies [16, 25] but aligns with a study conducted at a Nigerian university [28]. This disparity may reflect Nepal's patriarchal norms, where male students may feel additional pressure to succeed academically and secure a future career, given their expected role as primary earners. Further qualitative research is required to explore how gendered cultural norms shape stress experiences.

Similar to previous studies [22, 28, 29], no significant associations were found between academic stress levels and sociodemographic factors (age, religion, parental education, occupation, and family type). This indicates that academic stress is widespread across sociodemographic groups, although indirect factors, such as parental expectations and financial insecurity, may still play a role. Living arrangements showed descriptive variation in mean stress scores, but these differences were not statistically significant. In contrast, previous studies have shown that living without parents can increase stress levels [25]. The small sample size of students in nonfamily living arrangements likely limited statistical power, underscoring the need for larger mixed‐methods studies to clarify these relationships.

In this study, students with higher academic grades reported higher levels of stress. This finding aligns with previous research [8], indicating that high‐performing students experience additional stress due to pressure to maintain their academic status, which is often driven by internal motivations or expectations from parents and teachers. However, this difference was not statistically significant. Conversely, a study conducted in Karnali Province found that students with lower academic performance experienced greater stress [16]. This demonstrates a complex relationship between academic performance and stress, with factors such as academic struggles and pressure to maintain achievements potentially influencing stress levels.

A statistically significant difference in stress levels was observed on the basis of students’ self‐reported concern about their grades. Students expressing being “worried” or “very worried” had a lower mean stress score than those expressing being “not worried.” These findings imply that proactive academic performance might mitigate stress. Conversely, those who were less concerned about grades might experience higher stress due to unaddressed stressors, such as a lack of engagement and external pressures. However, this counterintuitive finding warrants careful interpretation and further investigation.

Furthermore, no significant differences in stress levels were found on the basis of self‐reported academic performance, ECA participation, or school environment perceptions. This highlights the potential significance of unmeasured factors, such as teaching quality, peer relationships (including bullying), household income instability, and individual resilience and coping strategies, which should be prioritized in future research.

## 5 Conclusion

Academic stress is highly prevalent among secondary students in Kathmandu, driven primarily by academic demands and workload. In this study of 353 secondary students in Kathmandu, 46.7% reported high academic stress levels. The dominant perceived stressors were workload and examinations, and male students reported significantly higher stress than female students. These findings highlight the need for school‐level interventions that reduce curriculum/examination pressures and strengthen academic counseling, study skills training, and coping support. Further longitudinal and qualitative studies are required to clarify the causal pathways and design targeted interventions.

## 6 Limitations and Strengths

This study has several limitations. First, using convenience sampling to select private schools limits the generalizability to public schools, rural settings, or other socioeconomic contexts. The geographic restriction to a single urban ward further narrows its applicability to Nepal's diverse educational and cultural landscapes. Although the PAS is a validated tool, stress measurement may be oversimplified by dichotomizing stress into high and low categories using a non‐validated cutoff. This cutoff aligns with prior studies, but its cultural and psychometric validity in Nepal remains unclear. The absence of an a priori sample size calculation may reduce the statistical power to detect subtle associations. Although the response rate was good, nonresponse bias remains possible, since absent students or those declining participation may differ from respondents. Finally, reliance on self‐reported data introduces potential biases, such as social desirability and recall inaccuracies, common in adolescent populations.

This study provides critical insights into academic stress in Kathmandu's competitive private‐school environment, despite its limitations. By employing a validated tool and achieving a high response rate, it reliably identified faculty workload and examinations as central stressors, revealing unexpected patterns, such as higher stress among male students and students who were unconcerned about grades. These findings underscore the urgency of addressing systemic pressures in Nepal's education system while highlighting gender‐related, culturally specific stressors. This study provides a foundation for policymakers to design targeted interventions, such as stress management workshops and equitable exam practices, to safeguard adolescent mental health in high‐pressure academic settings.

## Author Contributions

Rajesh Karki conceptualized the study design, performed the formal data analysis, and prepared the final manuscript draft. Anita Tamang conducted the literature review, developed and validated the research tools, managed the ethical approval process, and prepared the initial manuscript draft. Maheshor Kaphle supervised the study, contributed to data collection, and provided critical review and editing of the manuscript. All authors read and approved the final manuscript.

## Funding

The authors have nothing to report.

## Ethics Statement

Ethical approval was obtained from the Institutional Review Committee of Yeti Health Science Academy, Maharajgunj, Kathmandu, Nepal (Ref. No. 081/081‐412) on April 8, 2024.

## Consent

Written informed consent was obtained from the parents/guardians of all participants, alongside written assent from the adolescent participants. Data were collected anonymously, and participants were informed of their right to withdraw at any time without penalty.

## Conflicts of Interest

The authors declare no conflicts of interest.

## Data Availability

The data are available upon reasonable request from the corresponding author.
